# Can task-shifting work at scale?: Comparing clinical knowledge of non-physician clinicians to physicians in Nigeria

**DOI:** 10.1186/s12913-018-3133-7

**Published:** 2018-05-02

**Authors:** Manuela Villar Uribe, Olakunle O. Alonge, David M. Bishai, Sara Bennett

**Affiliations:** Johns Hopkins Bloomberg School of Public Health, Johns Hopkins University, Baltimore, MD USA

## Abstract

**Background:**

In contexts with severe physician shortages, the World Health Organization advocates task shifting to cadres with shorter training. To investigate the effects of task shifting at scale in primary health care, we assessed the clinical knowledge of non-physician clinicians versus physicians working in public primary care facilities in Nigeria.

**Methods:**

We assessed 4138 health workers using clinical vignettes of hypothetical patients suffering from illnesses commonly seen in primary care. Facility-level fixed effects models were used to compare health worker knowledge of (i) consultation guidelines, (ii) diagnostic accuracy and (iii) treatment guidelines.

**Results:**

Unadjusted averages of overall health worker knowledge were low across all types of worker except medical officers. After adjustment for potential confounding, the differences across all three measures between cadres became small or statistically insignificant.

**Conclusion:**

Non-physician clinicians can provide the same quality of primary care, for a set of common illnesses, as Medical Officers with similar personal characteristics, but clinical skills across cadres need strengthening.

## Background

A world-wide human resources for health crisis, which is particularly acute in Sub-Saharan Africa, has stimulated calls for further investment in human resource training and a search for alternative health service delivery models to fill large existing service delivery gaps [1–3]. The overall lack of trained health workers and difficulties in posting and retaining highly trained health workers to rural areas for the provision of primary health care, has led a number of countries to pragmatically shift their attention to the creation of new, or the strengthening of existing, non-physician clinician programs [4, 5]. With both lower training costs and times and greater retention rates in rural areas and within countries, non-physician clinicians are an important source of health care in many sub-Saharan African countries [4]. This shift has been supported by the World Health Organization (WHO) whose 2008 guidelines support task shifting to address health worker shortages [6].

Non-physician clinicians encompass a wide variety of cadres and can be broadly defined as health care providers that are neither physicians nor lay community health workers, but provide a broad spectrum of clinical care in the community, primary care facility or hospital [4]. Often called mid-level providers, this group of health workers includes clinical officers, medical or physician assistants, nurse clinicians or officers [4]. Commonly with post-secondary school training of 1–3 years, non-physician clinicians have been tasked with the delivery of basic primary care, minor surgeries, obstetrics [4, 7] and in some cases, more specific tasks such as the provision of antiretroviral therapy to HIV/AIDS patients [8], or the screening and management of patients with non-communicable illnesses [9].

The Nigerian health workforce is very large, comparable only to Egypt and South Africa in the region, however, the number of health workers is insufficient for its large population and they are inequitably distributed. Nigeria has over 56,000 doctors and nearly 225,000 nurses and midwives, with a population of approximately 170 million, this amounts to approximately 1.95 per 1000; far below the 2.5 per 1000 recommended by the WHO [2]. Primary care in the public sector is delivered in health centers and clinics, dispensaries and health posts that generally provide preventive, curative, promotive and pre-referral care. Primary level facilities are usually staffed with nurses, community health officers (CHOs), senior community health extension workers (CHEWs), junior CHEWs and environmental health officers [10].

As a response to a chronic shortage and an urban-rural maldistribution of physicians, since the 1970’s, Nigeria has implemented a wide reaching task-shifting strategy where non-physician clinicians have been trained to provide care in, and manage primary health facilities [11]. For school fees of about US$50–130 annually, CHOs, CHEWs and JCHEWs are trained by state-level schools or colleges of Health Technology [12] for four, three and two years, respectively, to deliver primary health care services [10] (see Table 1). Medical Officers, who also work in primary care facilities, on the other hand, are physicians with four to seven years of training whose schooling fees are of about US$300–500 per year.

**Table 1:** Nigerian Health Worker Cadre differences

	JCHEWs	CHEWs	CHOs	Nurse Officers	Nurse Midwifes	Medical Officers
Training	2 years	3 years	4 years	3 years	3 years +	4 years +
Roles	50% time in communities 50% time providing consultations	Consultations and facility management	General and supportive care	General and supportive care + midwifery care		General/family medicine
Facilities	Health Posts: Service Area: Village or neighborhood Target population: 500 Hours: 9 am-4 pm Services: Basic Curative Staff:					
	X	–	–	–	–	–
	Primary Health Clinics: Service Area: Villages or communities Target population: 2000–5000 Hours: 24 h Services: Basic + Normal Delivery Staff:					
	X	X	–	X	X	–
	Primary Health Centers (Ward Health Center): Service Area: Political Ward Target population: 10,000–20,000 Hours: 24 h Services: Basic + Basic Emergency Obstetric Care Staff:					
	X	X	X	X	X	X
Clinical Guidelines	National Standing Orders					Standard Treatment Guidelines
Approx. total fees for training	~US$100–260	~US$150–390	~US$200–520	~US$150–390		~US$1400–2000
Mo. Salary (Approx.)	~US$ 78	~US$104	~US$148	~US$165		~US$193

Source: World Bank (2010) [10] and National Primary Health Care Development Agency, Nigeria Federal Ministry of Health & WHO (2007) [39]

At the health facility, with the support of available nurses and lower-level health worker cadres, CHOs, CHEWs and JCHEWs give consultations, write prescriptions and perform basic treatments as guided by ‘National Standing Orders’ [13]. The National Standing Orders are clinical guidelines that act as a simple and accessible guide for non-physician clinicians in the identification and treatment of patients with common, basic illnesses, as would be provided by a fully trained physician [11]. The National Standing Orders give CHO’s, CHEWs and JCHEWs a legal backing for the management and treatment of the limited set of illnesses, commonly presented in a primary care setting [10]. In August of 2014, the Nigerian Federal Government approved a task-shifting (meant to move tasks to health workers with lower qualifications) and task-sharing (meant to develop workplace strategies for collaboration between health workers of different qualification levels) policy that has made official and further expanded the essential role of CHOs, CHEWs, JCHEWs and nurses in the delivery of primary care [14].

Available evidence from across the world suggests that non-physician clinicians can perform a number of specific tasks as well as physicians [5, 7, 9, 15–17]. There is, however, very little evidence on the ability of these mid-level cadres to identify and treat common illnesses at the primary care level as compared to physicians [18–21]. Further, many existing studies examine small-scale task-shifting projects, rather than nationwide implementation of the strategy. To our knowledge, there has not been a study that compares non-physician clinician knowledge or performance to that of physicians in Nigeria.

With this study we sought to compare the (i) knowledge of consultation process clinical guidelines, (ii) diagnostic accuracy, and (iii) knowledge of treatment guidelines for five common illnesses (diarrhea, pneumonia, diabetes mellitus, TB and malaria), between Medical Officers and non-physician clinicians who regularly deliver primary care in Nigeria. We defined non-physician clinicians as CHOs, Nurse Officers, Nurse Midwives, CHEWs and JCHEWs for this analysis. The null hypothesis of this study was of no inferiority in knowledge across the three outcome measures, of non-physician clinicians when compared to Medical Officers in Nigeria.

## Methods

### Sampling

This study uses the World Bank’s Service Delivery Indicators cross-sectional survey data collected from public sector, primary care facilities in twelve Nigerian states between July 2013 and January 2014 [22]. Using the official Federal Government list of public health facilities in Nigeria, facilities were stratified by state and urban/rural status. A total of 75–100 facilities were randomly selected from each strata for a total of 150–200 from each state. In each facility, health workers who reported providing outpatient consultations more than once per week were selected for the health worker knowledge interview. In facilities with less than 10 eligible health workers, all health workers present in the facility on the day of the survey were interviewed. In facilities with more than 10 eligible health workers, 10 health workers were randomly selected. A total of 4154 health workers from 2113 primary care facilities across 12 states were selected for the study, 16 (less than 1%) refused to participate.

This sample is representative of all public primary health care workers who regularly provide outpatient consultations in the 12 Nigerian states included in this study. Inverse probability weights were calculated, and used for each facility and individual health worker. Probabilities of selection were calculated to account for facility and health worker replacements where necessary.

### Assessing health worker knowledge

Health worker knowledge was assessed using clinical vignettes for seven simulated and standardized cases, where one enumerator acted as a patient presenting with a basic set of symptoms, and a second, recorded health worker questions, diagnoses, laboratory and treatment recommendations in a standardized questionnaire [18, 23–25]. All health workers were presented with the seven case simulations, face-to-face, with both enumerators present, in one session that lasted between 20 and 35 min. The standardized questionnaire included, for each case, a long list of questions or actions that could be mentioned by the health worker and their standardized answers designed to define an unambiguous clinical patient case. When a health worker posed a question, or action, the acting enumerator was trained to provide the standardized response given in the questionnaire, as if he or she were the patient, and the observing enumerator was trained to record a value of “1” if the predefined question or action were mentioned and a value of “2” if it were not mentioned by the end of the simulation.

The clinical vignettes used in this study were originally developed by a team of World Bank experts using WHO’s clinical guidelines for the selected illnesses, a clinical consensus on common presentations of these illnesses and were validated first in Senegal and Tanzania where the survey was first piloted [26]. The clinical guidelines were again reviewed and validated, to fit the Nigerian context and clinical guidelines, in 2013, with the use of local guidelines, expert consultations and pilot tests.

The five clinical vignettes are structured and delivered in a similar manner. Before the interview began, enumerators explained the interview process, recorded basic health worker information and performed a demonstration of a clinical vignette where one acted as the interviewer and the other as the health worker. The health worker was encouraged to ask any questions of clarification and provide their consent to proceed. For each hypothetical case, the enumerator, acting as the patient, presented him/herself, mentioning basic symptoms and the reason for seeking care. The pneumonia case for example, begins as follows: “Good morning (afternoon) doctor. I am the mother of this 5 year-old girl. Her name is Sia. She has a cough.” Following the introduction of the hypothetical case, the health worker asked any questions that are relevant for him/her to reach a diagnosis and treatment. The health worker can verbally perform a physical examination by asking such questions as the temperature, for which the standardized response would be “38.5 °C”. The questionnaire was designed for the “patient” enumerator to provide predefined answers to the health worker’s questions. All health workers were asked to give a diagnosis and recommend a treatment for each hypothetical case. The “observer” enumerator recorded all questions asked by the health worker for each hypothetical case. The questionnaire included the complete set of questions necessary to determine a presumptive diagnosis and treatment recommended as outlined in the national clinical guidelines. The questionnaire also included, approximately twice as many, commonly asked, non-essential questions that can be relevant or irrelevant to the diagnosis and treatment of the case.

As is outlined in Table 2, the five cases in the order they were presented to the health worker are: (1) a 13-month-old boy with acute diarrhea and severe dehydration, (2) a five year-old girl with pneumonia, (3) a 48 year-old man with type II Diabetes, (4) a 40 year-old man with Pulmonary Tuberculosis and (5) a four year-old boy with malaria and anemia. In an attempt to cover the breadth of knowledge required to deliver primary care, these five cases represent illnesses commonly seen by primary health care workers in Nigeria (and many other low- and middle-income countries), communicable and non-communicable illnesses of high public health concern as well as illnesses of children and adults.

**Table 2:** Vignette case definitions, essential questions and treatment as defined in National Standing Orders

Diagnosis-case presentation	National Standing Orders Definition
Acute diarrhea with severe dehydration- 13 month old boy with diarrhea	Severe condition Main symptoms: Frequent watery stool with weakness, with or without vomiting Examination: Lethargic or unconsciousness, sunken eyes, drinks poorly or unable to drink, skin pinch goes back very slowly Treatment: ORS by mouth if child can drink AND 100 ml/kg Ringer’s Lactate Solution, first 30 ml/kg in 30 min and then 70 ml/kg in 2.5 h. Reassess every 1–2 h.
Pneumonia- 5 year old girl with a cough	Moderate condition, suspect Pneumonia Main symptoms: Cough with fast breathing Examination: fast breathing (> 40 breath/min), no chest in-drawing, temp >37C, chest not clear, difficulty breathing (danger sign), inability to suck/drink (danger sign) Treatment: Cotrimoxazole 1 Tab (480 mg) bd ×5/7 OR Amoxicillin 250 mg/10mls qds × 5. Give Paracetamol if temp is > 37.5C, ½ tab or 250 mg qds × 5 days. Ask parent to bring child back in 2 days.
Diabetes Type II - 48 year old man feeling weak, without energy and is often hungry	Severe condition, suspect Diabetes Main symptoms: Passing a lot of urine, getting up more than 4 times at night to pass large quantities of urine but without pain, feel very thirsty and drinks a lot of water/fluid, loss of weight Examination: General weakness, examine urine for increased sugar and blood Treatment: Refer to hospital (in analysis, if Medical Officer: Oral Hypoglycemic or insulin when hypoglycemic are not effective)
Pulmonary Tuberculosis- 40 year old man suffering from fever and cough for some time	Moderate condition, suspect Pulmonary Tuberculosis Main symptoms: Cough of more than 3 weeks duration with or without chest pain with weight loss and shortness of breadth Examination: Coughing, sputum may or may not have blood, weight loss, breathlessness Treatment: Request for 3 sputum examinations, if at least 2 of 3 are positive, treat for pulmonary TB. Expectations of treatment depend on the receipt of specific TB training, not on specific cadres: if trained on TB management and have necessary drugs: First 2 months daily supervised: combined tablet of RHZE (150 mg + 75 mg + 400 mg + 275 mg). Daily for 6 months: combined tablet of EH(400 mg + 150 mg) OR supervised for 4 months: combined tablet of RH (150 mg + 75 mg). If not trained, refer to the Clinic.
Malaria with Anemia - 4 year old boy with fever for some time that is now worse	Severe Condition Main symptoms: Fever with any of the following: Vomiting, unable to feed, convulsions or history of convulsions Examination: Temp 37.5 C or above with any of the following: neck stiffness, change in alertness, convulsions, bulging fontanelle, moderate dehydration, lethargic Treatment: Continue feeding. Paracetamol 250 mg stat. Artemether-Lumefantrine 2 tablets twice daily for 3 days OR Artesunate+Amodiaquine 50 mg/135 mg one tablet once daily for 3 days. Oral Chloramphenicol 1.2 ml.

Source: NPHDA. 2010 [27]

All health workers were asked to complete all cases. Sixteen health workers that did not report their cadre and 27 health workers who did not complete all cases were excluded from this analysis (1% of the total sample): their characteristics were not significantly different from health workers who were included.

Health worker knowledge of the management of each case was assessed using the *Nigerian 2010 National Standing Orders for Community Health Officers and Community Health Extension Workers* [27], as the minimum desired standard of care. The three outcome measures of overall health worker knowledge of primary care practices were constructed in two steps. First, for each of the 5 cases, we generated three measures of knowledge: (i) consultation process clinical guidelines (a continuous variable for the % of essential history and physical examination questions asked), (ii) diagnostic accuracy (a dichotomous variable for correct/incorrect diagnosis) and, (iii) treatment guidelines (a dichotomous variable for full correct/incorrect treatment). In a second step, overall measures of (i) the percentage of essential questions in the clinical guidelines for the consultation process across the five cases, (ii) the percentage of five cases with a correct diagnosis and (iii) the percentage of five cases with a full correct recommended treatment were also calculated and used as the main outcome measures in this analysis.

Multivariate linear regression analyses were used to compare the knowledge of Medical Officers with that of non-physician clinician cadres, for each of the three overall knowledge outcome measures. Weighted ordinary least squares (OLS) regressions with facility-level clustered robust standard errors and facility-level fixed and random effects models were used in the analysis. The analysis tested the null-hypothesis of no inferiority in knowledge due to health worker training of non-physician clinician cadres as compared to Medical Officers. Based on existing literature (and variation across cadres in our sample), but restricted by available survey measures, we understood health worker knowledge as a function of individual health worker and facility-level characteristics. We included dummy variables for 10 different health worker cadres or general groups of cadres. Medical Officers are physicians with four of more years of clinical training and are used as our point of comparison as the standard of training for clinical care in a task-shifting strategy. Although included as individual dummy variables, we define non-physician clinicians as CHOs, CHEWs, JCHEWs, Nurse Officers and Nurse Midwives. We also included environmental health officers/assistants, community health assistants, health attendants/auxiliary nurses and dental officers/nurses/technicians, as a way to validate our measures, hypothesizing that these lower level cadres that have not received training in the delivery of primary care ought to perform less well in our measures of knowledge than the higher-level cadres.

Aside from health worker cadre (our independent variable of interest) we included controls for gender, years of experience as a health worker and a variable for the total number of non-essential questions the health worker asked across the five vignette cases. We found a high correlation (77.5%) between the variables of age and years of experience and chose to avoid multicollinearity in our models by including only years of experience, which we deemed to be theoretically more relevant: the variable was included as a spline at 8 years based on the distribution of our data. As an intrinsic characteristic, more outgoing and talkative health workers could naturally ask more questions and by chance ask more essential consultation questions as outlined in the clinical guidelines. Therefore, in an attempt to penalize guessing in the way we scored the vignettes we included, the total number of non-essential questions asked by the health worker across the five vignette cases. This represented the number of questions included in the questionnaire that the health worker asked, which were not essential to the consultation process, for the case, as outlined by the standing orders; these questions could be correct but unnecessary, or incorrect altogether. The most common of these non-essential questions concerned (i) as history taking, the current use of other medications, (ii) as part of the physical examination, the patient’s temperature and (iii) as a treatment, prescribing other or irrelevant antibiotics. Non-essential questions that were not included in the questionnaire were recorded as notes, however, unlike the non-essential questions listed in the questionnaire, we found these to be correlated with the individual enumerator undertaking the interview, and hence did not consider this as a reliable measure.

## Results

### Sample characteristics

We assessed the knowledge of a total of 4138 health workers that represent a population of approximately 42,000 health workers who regularly perform outpatient consultations at public primary health facilities in 12 Nigerian states (Table 3). We found that across the 12 states included in our sample, the vast majority (87.6%) of health workers that provide public primary care are non-physician clinicians, 2.6% are Medical Officers and 9.9% are lower-level cadres who have not been trained to provide this type of care. This weighted proportion of health worker cadres varies widely across states, however. Medical Officers represent 0.2% of this workforce in states like Bauchi and Taraba while representing over 25% in oil-rich Bayelsa. The proportion of non-physician clinicians varies between 69% in Bayelsa and 98% in the state of Niger. With some variation across cadres, on average, health workers in these 12 states are just over 40 years old, have 13.6 years of experience, are primarily female (71.6%), are posted to health centers (66.8%) as opposed to health clinics (23.4%) or health posts/dispensaries (9.8%) and just over half can be found working in rural areas (57.2%). Many of the provider characteristics could confound measures of health worker knowledge and were controlled for in multivariate analysis.

**Table 3:** Health Worker Sample Characteristics by cadre

	Medical Officer	CHO	Nurse Officer	Nurse Midwife	CHEW	JCHEW	Env Hlth Off/Ass	Comm Hlth Ass	Hlth Att/AuxNurse	Dental Off/Nur/Tech	Total
N	115	256	497	169	1891	802	110	93	168	37	4138
Wtd prop	2.6	5.9	12.7	4.7	44.2	20.1	2.6	2.2	4.4	0.7	100
Age (years)	40.5	45.0 ^a^	45.2 ^a^	36.8	40.4	35.7 ^a^	33.6 ^a^	41.1	39.1	34.9	40.5
(CI)	(36.3–44.7)	(43.7–46.3)	(44.1–46.2)	(34.7–38.9)	(39.9–40.8)	(34.9–36.4)	(32.1–35.2)	(39.3–42.9)	(37.6–40.7)	(31.3–38.6)	(36.3–44.7)
Experience (years)	13.6	20.7 ^a^	19.1 ^a^	9.2	14.9	10.5	7.9 ^a^	15.4	12.3	9.9	13.6
(CI)	(9.6–17.6)	(19.0–22.3)	(17.8–20.4)	(6.4–11.9)	(14.4–15.5)	(9.8–11.2)	(6.8–8.9)	(13.3–17.5)	(10.8–13.8)	(6.4–13.4)	(9.5–17.7)
Female (%)	20.7	62.3	86.4	99.8	71.2	65.4	37.6	81.4	89.1	70.9	71.6
Rural (%)	13.5	51.0	40.9	72.4	57.9	69.1	70.4	50.8	58.4	41.5	57.2
Total non-ess Q	63.1	37.5^a^	36.4 ^a^	37.3^a^	33.4^a^	32.7^a^	37.7^a^	25.1^a^	19.7^a^	36.8^a^	34.3^a^
(CI)	(57.0–69.2)	(35.0–40.2)	(34.6–38.3)	(34.2–40.4)	(32.4–34.4)	(31.2–34.3)	(32.0–43.4)	(21.2–28.9)	(17.8–21.6)	(29.3–44.4)	(33.6–35.0)
Facility Type											
Hlth Po/Di	3.0	6.5	2.4	3.9	10.9	12.0	17.8	20.6	15.4	7.7	9.8
Hlth Clin	35.0	15.4	20.7	10.6	26.0	28.0	22.1	11.6	6.7	36.0	23.4
Hlth Ctr	62.0	78.1	76.8	85.5	63.1	60.0	60.1	67.8	77.9	56.3	66.8
*(total)*	*100*	*100*	*100*	*100*	*100*	*100*	*100*	*100*	*100*	*100*	*100*
State											
Anambra	1.2	2.7	26.7	8.4	47.7	9.5	0.1	1.0	2.6	0.1	*100*
Bauchi	0.2	3.7	7.5	0.7	42.7	29.3	14.4	0.7	0.8	0.0	*100*
Bayelsa	25.9	9.6	8.7	9.6	28.4	12.4	0.2	0.2	0.3	4.8	*100*
Cross River	1.4	15.3	12.6	2.9	53.4	12.9	0.0	0.7	0.6	0.2	*100*
Ekiti	2.8	4.1	17.9	3.3	52.2	9.8	0.1	3.7	3.4	2.8	*100*
Imo	1.3	3.3	18.0	13.6	30.4	14.4	3.4	4.1	11.5	0.0	*100*
Kaduna	4.7	11.6	14.0	2.6	46.8	18.6	1.2	0.6	0.0	0.0	*100*
Kebbi	0.3	2.6	3.1	6.4	48.3	26.1	9.4	0.8	0.4	2.7	*100*
Kogi	0.8	7.0	12.4	0.0	53.6	21.1	0.4	3.0	1.0	0.8	*100*
Niger	1.5	2.4	4.8	1.8	51.7	37.2	0.0	0.1	0.6	0.0	*100*
Osun	4.0	7.5	17.2	2.6	31.8	11.8	0.0	6.7	17.4	1.0	*100*
Taraba	0.2	2.4	11.1	3.8	47.3	24.1	2.6	3.2	4.3	1.1	*100*

Note: Averages and proportions presented are weighted for the inverse probability of selection of each health worker across the different cadres. ^a^ t-test difference with Medical Officers *p* < 0.05

#### Cadre differences across outcome measures

Simple weighted mean comparisons of consultation process clinical guidelines, diagnostic accuracy and treatment guideline knowledge show low overall knowledge as well as significant differences between Medical Officers and non-physician clinicians. Medical Officers ask 1–2 (of approximately 4–6) more consultation questions as recommended by the clinical guidelines and are able to diagnose and adequately treat one more of the five cases presented (Table 4). Medical Officers on average, ask 56.8% of the recommended physical examination and history taking questions for a consultation, significantly more than CHOs (35.1%), Nurse Officers (35.7%), Nurse Midwives (34.3%), CHEWs (29.2%) and JCHEWs (27.2%). Similarly, Medical Officers accurately diagnose 72.2% of presented cases, significantly more than CHOs (53.3%), Nurse Officers (57.1%), Nurse Midwives (56.9%), CHEWs (46.4%) and JCHEWs (43.1%). The percentage of cases for which treatment was adequately prescribed, is much lower than the percentage of cases accurately diagnosed, for all cadres. Medical officers prescribed the full recommended treatment to less than half (43.5%) of presented cases, significantly more than CHOs (25.1%), Nurse Officers (27.0%), Nurse Midwives (26.2%), CHEWs (20.6%) and JCHEWs (19.3%). Across the three measures, lower level cadres without primary care consultation training; present lower scores than non-physician clinician cadres.

**Table 4:** Consultation Knowledge Outcomes by Case and Cadre

	N	Consultation Process^a^		Diagnosis^b^		Treatment^b^	
	*N*	*mean*	*CI*	*%*	*CI*	*%*	*CI*
Cadre							
Medical Officer	106	56.8	(50.6–63.0)	72.2	(64.6–79.7)	43.5	(36.3–50.8)
CHO	253	35.1^c^	(32.2–38.1)	53.3^c^	(48.2–58.3)	25.1^c^	(22.0–28.2)
Nurse Officer	494	35.7^c^	(33.6–37.8)	57.1^c^	(53.7–60.5)	27.0^c^	(24.3–29.8)
Nurse Midwife	168	34.3^c^	(30.8–37.8)	56.9^c^	(51.6–62.2)	26.2^c^	(20.7–31.7)
CHEW	1886	29.2^c^	(28.2–30.3)	46.4^c^	(44.9–47.8)	20.6^c^	(19.4–21.9)
JCHEW	799	27.2^c^	(25.9–28.4)	43.1^c^	(40.8–45.4)	19.3^c^	(17.5–21.1)
Env Hlth Off/Ass	110	26.0^c^	(22.1–29.8)	43.4^c^	(35.8–51.0)	18.1^c^	(13.0–23.3)
Comm Hlth Ass	92	19.8^c^	(16.1–23.5)	31.3^c^	(25.7–36.8)	13.8^c^	(10.0–17.7)
Hlth Att/AuxNurse	167	18.3^c^	(15.8–20.8)	30.3^c^	(26.7–33.9)	12.0^c^	(9.4–14.6)
Dental Off/Nur/Tech	36	24.0^c^	(17.6–30.4)	47.0^c^	(37.6–56.3)	21.9^c^	(16.6–27.2)
All	4111	30.1^c^	(29.4–30.8)	47.5^c^	(46.4–48.6)	21.7^c^	(20.8–22.6)

Note: Means presented here are weighted for the inverse probability of selection of each health worker

^a^The mean for consultation process is calculated as the average percentage history taking and physical examination questions asked by each type of health worker, across 5 cases

^b^ For each cadre, the % correct diagnoses and the %correct treatment is calculated as the average percentage of the 5 cases that was correctly diagnosed or treated by each type of health worker

^c^ t-test difference of outcome mean compared to Medical Officers *p* < 0.05

#### Regression models

**Fig. 1:**
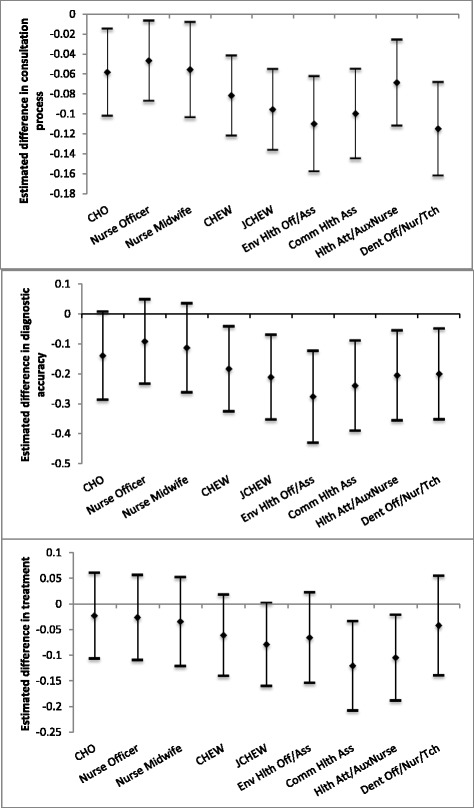
Estimated difference in consultation process, diagnostic accuracy and treatment knowledge across cadres as compared to Medical Officers. Graph A: Consultation Process; Graph B: Diagnostic Accuracy; Graph C: Treatment. Estimated values are adjusted for health worker gender, experience, number of non-essential question asked and facility-level characteristics. Zero value represents no difference when compared to Medical Officers. See Table 5 for specific values

The adjusted regression models show that non-physician clinicians have slightly but statistically significantly lower knowledge of the consultation process guidelines than Medical Officers. However, CHEWs and JCHEWs, but not CHOs, Nurse Officers and Nurse Midwives, have lower diagnostic accuracy than Medical Officers and all non-physician clinicians show an equal knowledge of treatment guidelines when compared to Medical Officers (Fig. 1). The sections below outline our regression model findings for each outcome.

#### Knowledge of consultation process clinical guidelines

After controlling for individual health worker and facility-level characteristics, we find that Medical Officers show slightly, yet statistically significantly, more knowledge of the consultation process clinical guidelines for primary care than non-physician clinicians (Table 5) and hence, rejecting the null hypothesis of no difference between cadres. On average, CHOs, Nurse Officers, Nurse Midwives, CHEWs and JCHEWs ask 5.8, 4.7, 5.6, 8.2, and 9.6% less recommended consultation questions than Medical Officers, respectively (model 1). We find that female health workers, but not less experienced health workers, show slightly but significantly lower knowledge of the consultation process guidelines than male health workers, while health workers who are more “talkative” (asked more non-essential questions) display significantly greater knowledge of the consultation process. As an important confounder, introducing the “talkativeness” variable in our regression models nearly eliminates the observed differences in consultation process knowledge between Medical Officers and non-physician clinicians. From our fixed-effects model we find that facility level effects account for 51.6% of the variation in the model, suggesting that the facility to which a health worker is assigned, has an important effect on their knowledge of the consultation process guidelines for primary care.

**Table 5:** Adjusted difference in consultation process, diagnostic accuracy and treatment knowledge across health worker cadres, compared to Medical Officers

	(1)	(2)	(3)
Variables	Consultation Process	Diagnostic Accuracy	Treatment
Medical Officer	[ref]	[ref]	[ref]
CHO	−0.0584^***^	− 0.139^*^	− 0.0222
	(0.0223)	(0.0750)	(0.0426)
Nurse Officer	−0.0468^**^	− 0.0914	− 0.0258
	(0.0205)	(0.0721)	(0.0423)
Nurse Midwife	−0.0557^**^	−0.113	− 0.0338
	(0.0244)	(0.0761)	(0.0442)
CHEW	−0.0817^***^	−0.183^**^	− 0.0603
	(0.0205)	(0.0726)	(0.0404)
JCHEW	−0.0956^***^	−0.211^***^	− 0.0784^*^
	(0.0207)	(0.0723)	(0.0412)
Env Hlth Off/Ass	−0.110^***^	−0.276^***^	− 0.0649
	(0.0243)	(0.0783)	(0.0451)
Comm Hlth Ass	−0.0998^***^	−0.239^***^	− 0.120^***^
	(0.0229)	(0.0769)	(0.0444)
Hlth Att/AuxNurse	−0.0688^***^	−0.205^***^	− 0.104^**^
	(0.0220)	(0.0767)	(0.0426)
Dent Off/Nur/Tch	−0.115^***^	−0.200^***^	− 0.0413
	(0.0239)	(0.0774)	(0.0495)
Gender			
Female	−0.0153^**^	−0.0160	− 0.0140
	(0.00670)	(0.0144)	(0.0123)
Experience			
< 8 yrs. experience	0.000986	0.00270	0.00313
	(0.00139)	(0.00283)	(0.00261)
8+ yrs. experience	−0.00185	−0.00383	−0.00288
	(0.00162)	(0.00321)	(0.00296)
Non-essential questions			
Total non-ess Q	0.00939^***^	0.0100^***^	0.00300^***^
	(0.000475)	(0.00101)	(0.000802)
Total non-ess Q^2^	−2.65e-05^***^	−3.72e-05^***^	1.52e-05^*^
	(4.34e-06)	(8.29e-06)	(8.78e-06)
Constant	0.112^***^	0.352^***^	0.132^***^
	(0.0244)	(0.0763)	(0.0435)
R-squared	0.559	0.259	0.185
Rho	0.5157	0.5950	0.5241
Observations	4040	4040	4040
Number of facilities	2086	2086	2086

Note: Facility-level fixed effects models. Values depict percentage point differences in knowledge of each outcome variable. Robust standard errors in parentheses ^***^ *p* < 0.01, ^**^ *p* < 0.05, ^*^ *p* < 0.1

#### Diagnostic accuracy

We find no statistical difference in the diagnostic accuracy of CHOs, Nurse Officers and Nurse Midwives, compared to Medical Officers, after controlling for health worker and facility-level characteristics (model 2) and hence, cannot reject the null hypothesis. The diagnostic accuracy of CHEWs and JCHEWs is significantly lower than that of Medical Officers, however. CHEWs and JCHEWs give a correct diagnosis to 18.3 and 21.1% fewer hypothetical cases than Medical Officers; this means they are able to diagnose approximately one case less (of 5) than Medical Officers are. As in the models for the knowledge of consultation process guidelines, we find that diagnostic accuracy increases with “talkativeness” of a health worker and as a strong confounder, its introduction in the model nearly eliminates the observed unadjusted differences between Medical Officers and non-physician clinicians. From the fixed effects model, we find that facility-level effects account for 59.5% of the variability in health workers’ diagnostic accuracy.

#### Treatment guidelines

We find no significant difference in the knowledge of treatment guidelines between Medical Officers and non-physician clinicians when controlling for health worker and facility-level characteristics (model 3) and hence, cannot reject the null hypothesis of no inferiority in knowledge of non-physician clinicians when compared to medical officers. Again, and more surprisingly, we find that knowledge of treatment guidelines increases as the number of non-essential questions asked increases, and its introduction in the model nearly eliminates the observed unadjusted differences between Medical Officers and non-physician clinicians. We find that 52.4% of the variation in the fixed effects model is due to facility-level characteristics.

To ensure the robustness of our models, we compared results across OLS, random and fixed-effects models. The OLS models, which were specified with the same covariates as the fixed- and random-effects models displayed very similar coefficients as the fixed- and random-effects models with equal statistical significance of coefficients with as other models. Hausman tests comparing fixed and random-effects models, suggest, only for the consultation process model that we cannot reject the possibility that facility level characteristics are in fact correlated with health worker characteristics. Comparison of means for each of the three outcomes across the three health facility levels in our model suggest that health workers in health clinics, followed by health centres, display higher (but not significant) knowledge as compared to health workers in health posts and dispensaries. The non-significant differences further suggest that facility effects likely arise from unmeasured facility characteristics. Differences across states and facilities further suggest the need for fixed effects models to control for unmeasured variables at the facility and state levels that could confound the correlation between health worker training and each of the knowledge outcomes. We also checked the robustness of our models by removing observations from the state of Bayelsa (the state with the highest proportion of Medical Officers), reaching similar findings. Additionally, we compared our models with equally specified models selecting only the top 2.6% performing non-physician clinicians for comparison with Medical Officers, consistently finding their ability to outperform these more highly trained health care workers across the three outcome measures. The comparison across models also suggests that the number of non-essential questions asked is a strong confounding variable throughout all models. The number of non-essential questions asked correlates with worker type (it is higher among medical officers but the relationship is not significant) and with quality of care score in the vignettes (it is correlated even within health worker cadre).

## Discussion

### Non-physician clinicians can provide the same quality of primary care as medical officers, but clinical skills across cadres need strengthening

Overall findings from this analysis suggest that task-shifting primary care to non-physician clinicians may not be detrimental to quality of service delivery. Our findings point to small, albeit significant differences in the knowledge of consultation process clinical guidelines between Medical Officers and non-physician clinician cadres, lower diagnostic accuracy for CHEWs and JCHEWs but not other cadres, and no significant differences in knowledge of treatment guidelines between these cadres, after controlling for gender, experience, the number of non-essential questions asked and facility-level characteristics.

Although often with limited health worker sample sizes, studies that have compared non-physician clinician performance in the delivery of primary care, to that of higher-level cadres have found mixed results [18–21, 28, 29]. In India, non-physician clinicians were found to display no significant differences in knowledge of primary care service delivery when compared to physicians [18] while health workers with shorter duration of training performed better in the provision of Integrated Management of Childhood Illness (IMCI) services than those with longer duration of training in Brazil and Uganda, had lower performance in Tanzania, but mixed results have been found in Bangladesh [20, 21]. Studies that have measured patient satisfaction with the provision of primary care have found that patients who received care from non-physician clinicians were equally [19, 29] or more highly [28] satisfied than those who received care from physicians.

Although we find only small, if any differences in knowledge between cadres, we find this within a context of low overall levels of knowledge. This is not the first study to find low health worker knowledge and adherence to clinical guidelines. Similarly to other studies that have assessed health worker knowledge of primary health care services using vignettes [18, 23, 30–34], our study finds low knowledge of clinical guidelines for the consultation process, low diagnostic accuracy and even lower knowledge of treatment guidelines. Studies that have compared levels of knowledge using vignettes to health worker performance in observed consultations, in low and middle-income countries, have found a “know-do-gap” that seems to show that health worker’s performance is consistently overestimated by their knowledge [25, 31, 33, 35, 36]. Other studies find that health workers with higher levels of training, in fact, exhibit less effort during a consultation than those with less training [24, 30]. In light of a potential know-do-gap, the widespread, low health worker knowledge points to even lower quality of primary care, than that measured here.

### It is important to consider the role of health worker “talkativeness” when undertaking assessments of knowledge using clinical vignettes

While unadjusted comparisons show that medical officers have substantially higher knowledge, these differences largely vanish when one adjusts for the number of non-essential questions asked which could indicate “talkativeness”. To our knowledge, this is the first study to explore the correlation of asking more consultation questions with health worker knowledge of primary care. We believe that this variable could represent either an intrinsic ability of the health worker or the use of guessing in the vignettes process. “Talkativeness” might enable health workers, of any cadre, to consider a broader number of potential diagnoses, lead them to ask more questions about the case and to rule out other possible diagnoses. On the other hand, this variable might also point to the use of a guessing strategy in the vignettes process. More research is needed to discern the relation of this factor on health worker knowledge.

### Facility-level factors affect health worker knowledge

We find, that facility-level factors explain close to half of the observed variance in performance. This is an encouraging finding because it suggests that facility level managerial interventions may improve quality. Comparable studies that assess health worker knowledge, in other contexts, have found less competent health workers in rural areas [24, 31, 37] and found differences across public and private facilities [33, 37]. More research will be necessary to understand the effects of specific facility characteristics on health worker knowledge.

Non-physician clinicians already provide the large majority of primary health care services in Nigeria, and the government’s 2014 policy on Task Shifting and Sharing is further emphasizes the role of NPCs in the health system. Our findings indicate that this is unlikely to negatively affect the quality of care provided, and, given the lower cost and shorter training period of NPCs the policy will improve the use of scarce health care resources. We also know from other studies [4] that NPCs are more likely to come from, and be willing to work in rural and remote areas than physicians thus also improving access to services among those in most need. Our findings therefore fully support the existing policy direction in Nigeria, but they also point to an urgent need to strengthen the clinical skills of both physicians and non-physicians.

A limitation of this study may be the use of the *National Standing Orders* as the minimum desired standard of care. These guidelines are used for training CHO, CHEWs and JCHEWs but not Nurses and Medical Officers. Hence, using the standing orders may have introduced a measurement error, and contributed to the finding that Medical Officers asked significantly more non-essential questions, which may be appropriate for their relative higher level of training. However, since the goal of our study was to understand the comparative knowledge of health workers for delivery quality care, irrespective of the training standards, this is unlikely to have affected our conclusions. A limitation of the data collection methodology is its assessment only of knowledge and not effort in service provision. Even though the clinicians interviewed in the study did not have time to prepare for the assessment, it is likely that the interview process created a Hawthorne effect where participants meant to exhibit their greatest level of knowledge. As discussed above, other studies have shown that health workers tend to perform better in knowledge assessments than in cases when assessed by standardized patients. The data collection methodology hence limiting conclusions to only what health workers know and not what they actually do in practice. Lastly, a limitation of this study is our lack of information on the formal or informal re-training of the health workers who were interviewed, the availability of this information would have allowed us to better discern the importance of in-work for the improvement of health worker knowledge in service delivery.

## Conclusion

Within a context of low overall knowledge, our findings suggest that non-physician clinicians in Nigeria have similar knowledge of clinical guidelines, more highly trained cadres (but not CHEWs and JCHEWs) have equal diagnostic accuracy and all cadres have equal knowledge of treatment guidelines when compared to physicians after adjusting for “talkativeness”. The number of non-essential questions asked by a health worker is a strong confounder for each of the three knowledge outcomes, and helps explain much of the observed unadjusted differences in knowledge between cadres. The results suggest that a health worker’s “talkativeness” is, together with their level of training, and facility where they are posted, essential to their performance on assessments of knowledge of primary care and key to task-shifting strategies.

Our findings highlight the overall low level of health worker knowledge in Nigeria, reinforcing the findings of studies from other low- and middle-income countries. Lack of knowledge of diagnostic and treatment guidelines is likely to lead to even lower levels of health worker performance. Systematic reviews have established that short courses and in-service training alone are rarely effective in strengthening health worker skills [38]. In order to address this challenge in Nigeria, there is clearly a need for multifaceted efforts encompassing interventions to strengthen pre-service and in-service training, as well as facility level interventions to support better supervision and performance management, and more effective approaches to health worker recruitment and selection.

## Abbreviations

CHEW: Community Health Extension Worker
CHO: Community Health Officer
HIV/AIDS: Human Immunodeficiency Virus/Acquired Immune Deficiency Syndrome
IMCI: Integrated Management of Childhood Illness
JCHEW: Junior Community Health Extension Worker
OLS: ordinary least squares
WHO: World Health Organization
